# The HPQ—Development and First Administration of a Questionnaire for Hypoparathyroid Patients

**DOI:** 10.1002/jbm4.10245

**Published:** 2019-11-07

**Authors:** Deborah Wilde, Lara Wilken, Bettina Stamm, Martina Blaschke, Christina Heppner, Mira‐Lynn Chavanon, Andreas Leha, Christoph Herrmann‐Lingen, Heide Siggelkow

**Affiliations:** ^1^ Clinic of Gastroenterology and Gastrointestinal Oncology University Medical Center Goettingen Goettingen Germany; ^2^ ENDOKRINOLOGIKUM Saarbrücken Saarbrücken Germany; ^3^ MVZ Endokrinologikum Goettingen Goettingen Germany; ^4^ Department of Psychosomatic Medicine and Psychotherapy University Medical Center Goettingen Goettingen Germany; ^5^ Institute for Medical Statistics University Medical Center Goettingen Goettingen Germany

**Keywords:** HYPOPARATHYROIDISM, QUALITY OF LIFE, QUESTIONNAIRE, SYMPTOM SCALE

## Abstract

Hypoparathyroidism patients suffer a variety of complaints often leading to reduced quality of life. Currently, no specific standard instrument exists to measure corresponding disease manifestations. We therefore aimed to develop a disease‐characteristic questionnaire for hypoparathyroid patients. We used an analytical‐empirical approach for questionnaire construction based on retrospective analysis of four well‐established but non‐disease‐specific questionnaires (Symptom Checklist 90, revised [SCL‐90‐R]; Giessen Complaint List [GBB]; Short‐Form‐36 Health Survey [SF‐36]; von Zerssen Symptom List [B‐L Zerssen]) and two additional unpublished or local questionnaires (SHGdQ and GPQ) in a German hypoparathyroidism self‐help group (*n* = 60). Retrospective data were compared with corresponding general population norms. The new questionnaire was administered prospectively over 1 year to patients with postoperative hypoparathyroidism and two control groups to validate specificity. Exploratory factor analysis (EFA) and reliability testing were applied to identify relevant scales and reduce overlapping items. In the self‐help group, SCL‐90‐R revealed elevated symptom load in four complaint areas (*p* = 0.003 to *p* < 0.001). The SF‐36 mental summary score (*p* < 0.001) and further scales were lowered. In the GBB, four of five scales (*p* = 0.009 to *p* < 0.001) were elevated. In the B‐L Zerssen, 6 of 24 items revealed complaint areas. Based on these findings, the new 40‐item “Hypoparathyroid Patient Questionnaire” (HPQ 40) was developed, tested prospectively, and further analyzed. EFA revealed five scales (pain and cramps, gastrointestinal symptoms, depression and anxiety, neurovegetative symptoms, loss of vitality), all with Cronbach's alpha >0.7. The questionnaire was revised accordingly and shortened to 28 questions to avoid redundancy. We present a new disease‐characteristic questionnaire for hypoparathyroidism patients. Prospective testing revealed five major complaint areas and promising psychometric properties. This questionnaire can be tested for usefulness in further clinical trials. © 2019 The Authors. *JBMR Plus* published by Wiley Periodicals, Inc. on behalf of American Society for Bone and Mineral Research. © 2019 The Authors. *JBMR Plus* published by Wiley Periodicals, Inc. on behalf of American Society for Bone and Mineral Research.

## 1 Introduction

Hypoparathyroidism is an endocrine disease characterized by low serum calcium levels due to insufficient parathyroid hormone (PTH) secretion mostly resulting from damage to or removal of the parathyroid glands during thyroid or neck surgery. Less common causes of hypoparathyroidism are autoimmune or genetic in nature.^1^

Because recombinant human PTH (rhPTH 1–84) substitution has only become available recently and is restricted to special clinical conditions, the current standard treatment comprises combining active vitamin D agents with oral calcium supplementation. However, this treatment is unable to restore the normal physiology of calcium homeostasis. Furthermore, patients suffer from renal complications as well as soft tissue calcifications.^2^ In addition to the typical clinical symptoms of hypocalcemia (eg, muscle cramps, tingling in the extremities, and perioral numbness), neurocognitive and psychologic impairment has been described in hypoparathyroid patients.^3^, ^4^ Moreover, an increased risk of developing cataracts, cardiovascular disease, and infections has been described.^5^, ^6^ Impaired muscle function was reported by one and reduced quality of life by several studies.^7^, ^8^, ^9^

Although European guidelines and a guidance paper on disease control regarding laboratory results are now available,^10^, ^11^ there is still no gold standard in the assessment of patients’ subjective symptoms in clinical practice.

Several methods have been used to characterize the disease symptoms and clinical manifestations. An online survey was conducted and different nonspecific questionnaires (eg, SCL‐90‐R, SF‐36, Hindi Mental State Examination) were applied to measure current complaints or quality of life quantitatively.^3^, ^4^, ^7^, ^12^ Because online surveys are not particularly practicable in daily clinical routine and the concurrent use of a number of different validated questionnaires to cover all patients’ symptom domains is time consuming, the development of a designated instrument is bound to be of further value. In addition, it remains unclear as to whether generic questionnaires address all the aspects important to a certain disease.^13^ Therefore, a questionnaire designed to address a specific disease may not only cover domains more relevant to that condition but may also be more sensitive to changes over time.^14^

In this study, our aim was to develop an instrument targeting patients with hypoparathyroidism to measure the most common and relevant symptoms in this disease and test its clinical usefulness as well as psychometric properties.

## 2 Subjects and Methods

### 2.1 Design

**Figure 1:**
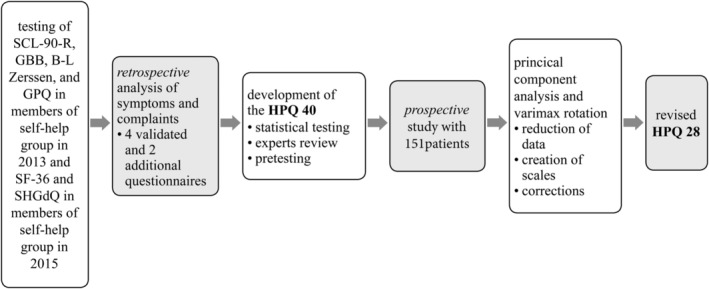
Summary of the study design. Retrospective part with analysis of six questionnaires in total (SCL‐90‐R, GBB, B‐L Zerssen, GPQ, SF‐36, self‐help‐group‐derived questionnaire [SHGdQ]), development and prospective testing of the HPQ 40, and revision to HPQ 28.

The development of the disease‐characteristic 40‐item hypoparathyroid patient questionnaire or HPQ 40 was divided into a retrospective and a prospective element. Fig. 1 provides an overview of the developmental process.

Three validated questionnaires (Symptom Checklist 90 [SCL‐90‐R], Giessen Complaint List [GBB], von Zerssen Symptom List [B‐L Zerssen]) as well as the general patient questionnaire (GPQ) from the community health center “MVZ Endokrinologikum Goettingen” were filled in by members of a German hypoparathyroidism self‐help group during their meeting in 2013 to explore patients’ symptoms. Although not generally being instructed to do so, patients commented on different questionnaires. These patient comments were collected systematically, reworded into questions, and then renamed as the so‐called “self‐help‐group‐derived questionnaire” (SHGdQ). This questionnaire was administered together with the 36‐Item Short Form Health Survey (SF‐36) in the patients’ self‐help‐group annual meeting in 2015. Hence, the questionnaires administered in 2013 were different from 2015 for all participants. With regard to the patients, there was probably an overlap, since they were all members of the self‐help group. However, data were collected anonymously by using numbers to identify the connected questionnaires. It follows that no analysis of the overlap was possible. Data were analyzed according to certain criteria, which are summarized in Table 1. First, items were identified by their median or the percentage of positive responses. Second, the items were included in the new questionnaire if they belonged to a scale that significantly differed between patients and population norms. In addition, items were included if two or more items identified by their increased median/percentage described the same content, eg, “how much of the time … did you feel tired? – A good bit of the time” (SF‐36) and “tiredness” (GBB) emphasizing the relevance of tiredness in general. In the following, items with similar meaning were combined and reworded for the new questionnaire.

**Table 1:** Steps and Criteria Applicable When Developing the New HPQ 40 From Several Generic Questionnaires (SCL‐90‐R, SF‐36, GBB, B‐L Zerssen, SF‐36)

1. Considering all items with	
Questionnaire	Criteria
‐ SCL‐90‐R	Median ≥1
‐ SF‐36	Median adapted to changing polarity of scales
‐ GBB	Median ≥2
‐ B‐L Zerssen	Median ≥2
‐ General patient questionnaire	Percentage of “yes” answers ≥40
‐ Additional questionnaire	Median ≥2
2. Selection of items on a significant scale in comparison with general population norms	
3. Combination of items with same meaning (analogy) and new wording	

All items identified by the criteria listed in Table 1 were adapted from the generic questionnaires, rephrased when needed, and then structured as the preliminary disease‐characteristic HPQ 40. This version was reviewed in terms of structure and completeness by an endocrinologist as well as a panel of psychologic experts. This review resulted in the inclusion of two established screening items for depression (namely the PHQ‐2 screening questions) as two of the 40 items for internal control of those items possibly characterize depressive symptoms. The resulting final version of the HPQ 40 was tested on a small group of healthy adults with respect to understanding and time required to complete the questionnaire.

As next step, the HPQ 40 was tested again on hypoparathyroidism (hypoPT) patients. To assess the characteristic symptoms of a disease once it has been diagnosed and to monitor the intensity during the course of the disease, any comparison with a normative control is neither commonly accepted nor conducive. As described above, we actually compared our patients with population samples to identify items of potential relevance to hypoPT patients in the first step. In the subsequent step, it appeared to be far more useful to compare our hypoPT patients with control groups suffering from other diseases in order to demonstrate that the HPQ 40 does not simply respond to illness in a nonspecific manner.

Hence, the new disease‐characteristic HPQ 40 questionnaire was prospectively tested on 65 patients with chronic postoperative hypoPT as well as 49 patients who had undergone thyroid surgery without hypoparathyroidism (ThySu), and 37 patients with current or former hyperparathyroidism (PHPT). The ThySu control group was chosen on the basis of an identical surgical procedure as in hypoPT patients. The PHPT group as control represented another parathyroid disease also presenting a change in calcium levels.

The study was approved by the Ethics Committee (IRB) of University Medical Center Goettingen (no. 25/10/15); all subjects provided written informed consent before prospective participation. Data were pseudonymized. All questionnaires were administered, filled out, and evaluated in German.

### 2.2 Patients

The characteristics of all the study groups are presented in Table 2.

**Table 2:** Patient Characteristics for the Different Study Groups – Age (Mean ± SD) and Sex (*n* [%])

		SHG in 2013	SHG in 2015	hypoPT	PHPT	ThySu
Age		52 ± 10	54 ± 14	57 ± 10	58 ± 18	51 ± 16
Sex	Male	4 (12)	5 (19)	14 (22)	6 (16)	5 (10)
	Female	29 (88)	22 (81)	51 (78)	31 (84)	44 (90)

SHG = Self‐help group; hypoPT = hypoparathyroidism; PHPT = primary hyperparathyroidism; ThySu = thyroid surgery.

All subjects in the self‐help group were members of the *Network Hypopara*, a German patient organization. The subjects of the hypoPT, ThySu, and PHPT groups were recruited from two different endocrinologic centers in Goettingen and Saarbruecken, Germany. Patients for prospective testing were identified from their medical records. Postoperative hypoparathyroidism was diagnosed on the grounds of laboratory findings in combination with clinical symptoms, as there is no international standard definition for hypoparathyroidism to date.

### 2.3 Questionnaires used in the retrospective study

The SCL‐90‐R is a self‐reported symptom inventory with 90 items covering several dimensions of psychopathology.^15^ It can be divided into nine scales (interpersonal sensitivity, depression, somatization, obsessive–compulsive, phobic anxiety, paranoid ideation, hostility, anxiety, and psychoticism) as well as one global severity index (GSI). Items require patients to record their feelings during the last 7 days and can be answered on a five‐step scale from 0 = “not at all” to 4 = “extremely” and averaged across each subscale.

The GBB consists of 57 items^16^ that are reduced to 24 items in its short form GBB 24, which is further described here. Questions are structured in four symptom domains (gastric symptoms, pain in the limbs, exhaustion, and heart complaints). Furthermore, a global score of bodily discomfort (GSD) can be calculated. Answers are given on a scale from 0 to 4 with higher values indicating higher symptom load.

The B‐L Zerssen^17^ comprises 24 questions that can be rated on a scale from 0 to 3 and then transformed to a global score that describes the overall bodily or general discomfort. Values for the global score are transformed to *T*‐scores (mean = 50, SD = 10), where a *T*‐score ≥ 60 is regarded as elevated.

The SF‐36 is an often‐used survey tool for health‐related quality of life.^18^, ^19^ Items represent eight dimensions of either physical or mental health (physical functioning, physical role functioning, vitality, social functioning, bodily pain, general health perception, emotional role functioning, and mental health). Furthermore, a physical as well as mental component scale can be calculated. Scores range from 0 to 100 and higher scores represent a better quality of life.

Values resulting from these four validated questionnaires and their scales can be compared with published norms for the general population. None of these questionnaires is specific to any disease; they are used for more general purposes.

The general patient questionnaire (GPQ) from “MVZ Endokrinologikum Goettingen” contains 69 mostly binary questions representing the 15 domains pain, gastrointestinal symptoms and disease, neurologic symptoms, cardiovascular symptoms and disease, blood and vascular disease, renal disease, hormonal dysfunction, musculoskeletal disease, depression, substance addiction and food, psychologic problems, trauma, and finally unspecific symptoms. This GPQ is filled in by every patient presenting in the “MVZ Endokrinologikum Goettingen” during their first visit to facilitate taking the patient medical history. The self‐help‐group‐derived questionnaire (SHGdQ), tested prospectively in 2015, contained symptoms added in writing by patients during the previous questionnaires. Some questionnaires, for example the GBB, include sections for free comments on additional complaints. Furthermore, patients simply commented on questionnaires, although not being instructed to do so. We interpreted this as evidence that common questionnaires may not adequately depict all specific symptoms and therefore the need for additional comments was great. Hence, all patients’ comments were collected systematically and evaluated by several persons with respect to their meaning. Any symptom mentioned by at least two different patients was included in this particular questionnaire for reassessment. Answers were possible on a scale from 0 = “not at all” to 4 = “very strong.”

### 2.4 Statistics

Data were collected and analyzed with IBM's (Armonk, NY, USA) SPSS software package in versions 22 and 24. Answers for all questionnaires were coded from 0 to 3 or 4 depending on the respective questionnaire scales employed, where 0 was the lowest response option (eg, 0 = “not at all”) and 3 or 4 the highest (eg, 4 = “severely”). In the retrospective section of the study (the first step), patients’ values on the SCL‐90‐R, GBB, B‐L Zerssen, and SF‐36 were compared with general population norms. Group differences were evaluated using either the Student's *t* test (for one or two independent samples) for parametric data or the Wilcoxon test for nonparametric data. Data are presented as mean ± SD or median (25% percentile; 75% percentile). Alpha level was set at *p* = 0.05 and we applied Bonferroni correction for multiple testing. Items of the general patient questionnaire and the self‐help‐group‐derived questionnaire were analyzed considering the percentage of positive responses or median (please also refer to Table 1).

In the subsequent step, prospective data from hypoPT patients compared with the two control groups using the HPQ 40 were evaluated using principal component analysis (PCA) and varimax rotation as part of an exploratory factor analysis (EFA), in order to identify relevant factors that may represent patients’ specific symptom domains. PCA is a dimension‐reduction tool that may be employed to reduce a large set of variables (in this analysis, the 40 items of the HPQ 40) to a smaller set without losing information. A varimax rotation is an orthogonal rotation mostly used in factor analyses to maximize the variance between factors; thus high factor loadings become even greater and small factor loadings become smaller. It is a standard statistical procedure to improve factor evaluation.

In this study, the statistical term factor correlates to the term scale, which relates to symptom‐specific groups. The scales were named accordingly to define blocks of questions with a common symptom description and/or background. Items with factor loadings ≥0.5 were included. If an item loading was >0.5 on more than one factor, that item was allocated to the factor/scale with the greatest correlation. As an example, diarrhea was allocated to the scale gastrointestinal symptoms and to the scale neurovegetative symptoms (NVS). The correlation to the scale NVS was greater; therefore, the item was now reallocated to the scale NVS. The factors found were regarded as the different patients’ symptom scales. Under consideration of Cronbach's alpha >0.7, scales were shortened or extended to ensure the reliability of the scale. Cronbach's alpha is a measure of internal consistency, reflecting how closely related a set of items are as a group. Ideally, Cronbach's alpha should range between 0.70 < α < 0.90. In addition, reliability was also measured by correlations between an item and the remaining items in the scale (corrected item‐scale correlations). The final five scales identified covered 23 items of the 40‐item questionnaire. All items that were not part of any identified scale were analyzed for group differences with Kruskal‐Wallis tests. Those items that did not differ significantly between the different patient groups (hypoPT, ThySu, and PHPT groups) or did not qualify for the above‐mentioned reasons were withdrawn from the revised version.

Scores on the identified symptom scales were obtained by calculating the mean value of the items belonging to the scale for each patient. In doing so, high scores indicate great impairment, low scores low impairment. We screened for depression by summing up values for the two screening items.^20^ Values ≥3 indicated positive screening.

## 3 Results

### 3.1 Retrospective analysis

**Figure 2:**
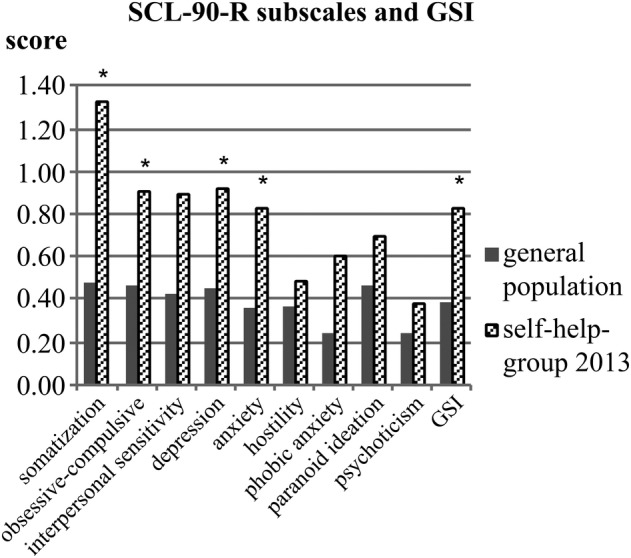
Scores of the SCL‐90‐R scales (somatization, obsessive–compulsive, interpersonal sensitivity, depression, anxiety, hostility, phobic anxiety, paranoid ideation, psychoticism) and the global severity index (GSI) of the self‐help group in 2013 in comparison with the general population norms. *Significant difference (*p* < 0.05).

Analysis of the SCL‐90‐R, depicted in Fig. 2, revealed significantly greater complaints for the self‐help group when compared with the general population norms (GPop) with regard to somatization (self‐help group: 1.32 ± 0.91 versus GPop: 0.47 ± 0.47, *p* < 0.001), obsessive–compulsive symptoms (0.91 ± 0.83 versus 0.45 ± 0.47, *p* = 0.003), depression (0.92 ± 0.78 versus 0.44 ± 0.51, *p* = 0.001), and anxiety (0.82 ± 0.85 versus 0.34 ± 0.45, *p* = 0.003). Patients also had a higher global severity index score (GSI; 0.82 ± 0.7 versus 0.38 ± 0.39, *p* = 0.001). No significant group differences (after Bonferroni correction, *p* < 0.006) were detected for interpersonal sensitivity, hostility, phobic anxiety, paranoid ideation, or psychoticism. As the self‐help group consisted mainly of women (>80%), a separate analysis for women and their reference population was conducted, revealing the same significant differences as for the whole group (data not illustrated).

**Figure 3:**
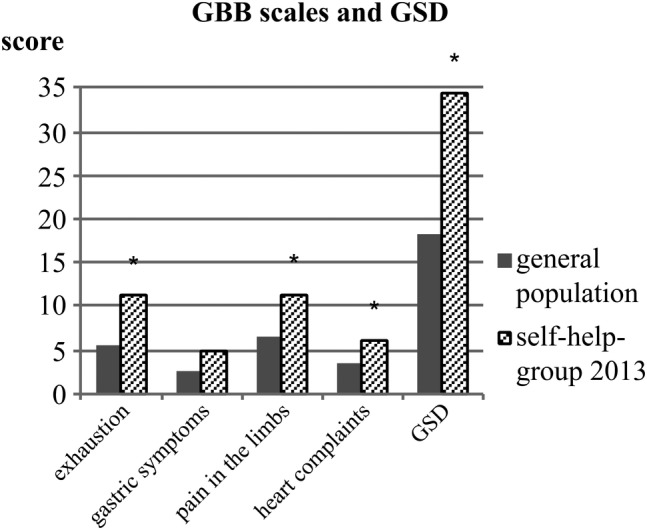
Scores of the GBB scales (exhaustion tendency, gastric symptoms, pain in the limbs, heart complaints) and global score of discomfort (GSD) for patients of the self‐help group in 2013 in comparison with the general population norms. *Significant difference (*p* < 0.05).

In comparison with the reference population (GPop), the self‐help‐group results on the GBB differed in four of five domains (Fig. 3). Patients scored significantly higher on the subscales pain in the limbs (self‐help group: 11.36 ± 6.4 versus GPop: 6.51 ± 4.94, *p* < 0.001), exhaustion (11.17 ± 6.78 versus 5.55 ± 4.62, *p* < 0.001), heart complaints (6.07 ± 5.11 versus 3.41 ± 3.68, *p* = 0.009), and global score of discomfort (GSD; 34.17 ± 19.5 versus 18.18 ± 13.46, *p* < 0.001), whereas (after Bonferroni correction) scores on gastric symptoms did not differ significantly. In accordance, the B‐L Zerssen exhibited a significantly elevated *T*‐score (*T* = 61.55).^21^

**Figure 4:**
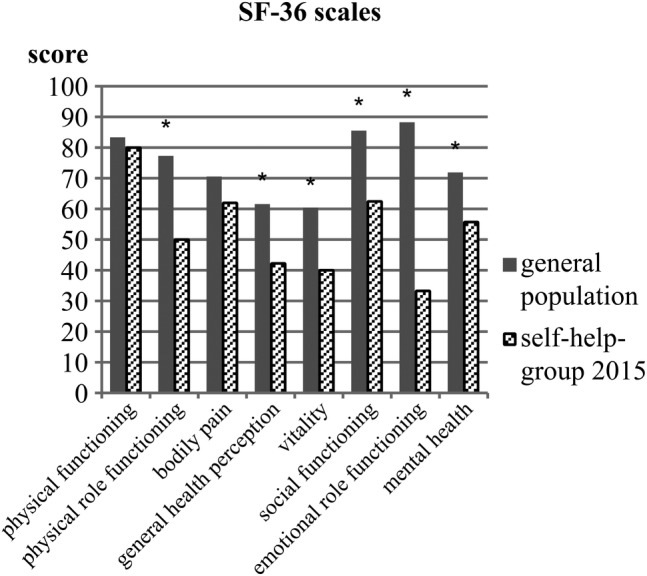
Scores of the SF‐36 scales (physical functioning, physical role functioning, bodily pain, general health perception, vitality, social functioning, emotional role functioning, mental health) of the self‐help group in 2015 in comparison with the general population norms. *Significant difference (*p* < 0.05).

Values for the SF‐36 in the self‐help group were significantly lower compared with age‐ and sex‐matched healthy controls for physical role functioning (presented as median [with 25%; 75% percentile]; self‐help group: 50.00 [00.00; 100.00] versus GPop: 77.81 [72.34; 86.91], *p* = 0.004), general health perception (43.50 [18.75; 57.00] versus 61.08 [58.92; 68.14], *p* < 0.001), vitality (40.00 [35.00; 50.00] versus 60.01 [58.29; 62.34], *p* < 0.001), social functioning (62.50 [0.00; 100.00] versus 85.30 [85.30; 87.72], *p* = 0.004), emotional role functioning (33.33 [00.00; 83.33] versus 88.05 [88.05, 89.99], *p* < 0.001), and mental health (56.00 [48.00; 64.00] versus 71.11 [70.22; 71.81], *p* < 0.001), indicating a greater impairment in these areas (Fig. 4). Furthermore, the mental component scale was also significantly lowered (self‐help group: 36.04 ± 9.81 versus GPop: 51.54 ± 8.14, *p* < 0.001). Regarding physical health, neither the physical component scale nor the physical functioning or bodily pain scale revealed any significant impairment in hypoparathyroid patients when compared with general population norms.

The following items from the GPQ were identified as relevant according to the percentage of “yes” answers ≥40 (percentage in parenthesis): concentration disturbance (73), exhaustion tendency (70), sleep disturbance (58), back pain (55), sweating (50), neck pain (46).

With a median ≥2, the “self‐help‐group‐derived questionnaire” revealed several muscle complaints (eg, cramps), tingling, and reduced function in private and everyday life as important features.

### 3.2 Prospective trial with development of the new questionnaire

From a total of more than 150 questions from all six questionnaires, relevant items were filtered applying the previously explained criteria as illustrated in Table 1. A four‐step scale (0 = “not at all,” 1 = “slightly,” 2 = “moderately,” 3 = “severely/strongly”) was selected to express the intensity of symptoms. After expert review, two screening questions for depression (from the PHQ‐2), one question on bodily health, and two gastrointestinal items (nausea and upset stomach, abdominal pain, or stomach cramps) were added to the new HPQ, which finally contained 40 items and was referred to as the HPQ 40. The final selected items were arranged in three blocks: the first and largest contained 32 negatively phrased items; the second consisted of six positive items; the last one contained the two screening questions for depression from the PHQ‐2, for which the established scale for frequency (0 = “not at all,” 1 = “on single days,” 2 = “more than 50% of the time,” 3 = “almost every day”) was applied.

When analyzing the data, it was important to invert the scaling for the six positively formulated questions, so that high numbers also reflected high symptom load corresponding to impaired vitality.

An appropriate design was created to structure the questionnaire visually and to arrange all items on one page. The HPQ 40 was then pretested on a group of healthy individuals resulting in corrections to the format and spelling. Test persons claimed no difficulties with understanding, and the time required to complete the test was around 5 minutes.

### 3.3 First results of the HPQ 40: exploratory factor analysis and scales

In the subsequent step, it was far more useful to compare the hypoPT patients with control groups of patients suffering from other diseases to demonstrate that the questionnaire does not simply respond to illness in a nonspecific manner. Hence, we prospectively tested the HPQ 40 on the hypoPT, ThySu, and PHPT patient groups.

One main task of the following analysis was to determine whether groups of questions (and thereby symptoms) can be identified. With these groups of questions, differences between the hypoPT patients and the control groups could then be analyzed. In the subsequent statistical analysis, the groups of questions were termed “symptom domains,” “factors,” or “scales,” and the single questions are referred to as “items.”

The following process evaluated the relevant symptom domains as preliminary scales of the test. Through PCA and varimax rotation, a total number of five new factors (symptom domains, further termed as “scales”) were identified as maintaining a high level of explained variance (>50%). These five scales were defined according to the wording of the corresponding items: pain and cramps (PaC), gastrointestinal symptoms (GIS), depression and anxiety (DaA), neurovegetative symptoms (NVS), and loss of vitality (loss of VIT). The 23 items of these five scales as well as the loadings and Cronbach's alphas are presented in Table 3. As the DaA scale initially comprised 12 items, it was shortened to five under consideration of Cronbach's alpha to avoid redundancy. Cronbach's alpha for NVS was slightly below 0.7, but adding the item “In the past 4 weeks, how much did you suffer from a sense of weakness?” to the scale increased its value to 0.737, which made it reasonable to maintain this item in the NVS scale, despite its loading of slightly below 0.5. The corrected item‐scale correlation as additional parameter of internal consistency is presented in Table 3. All values calculated were above 0.4.

**Table 3:** Scales of the HPQ 40/28 and Their Cronbach's Alpha Values as Well as Items and the Items’ Correlation With Their Factor (Scale) in EFA (=Loading)

Scale	Cronbach's alpha*	Items	Loading	Corrected item‐total correlation
Depression and anxiety	0.860			
		Self‐blaming emotions	0.79	0.665
		Inner tension and restlessness	0.71	0.696
		Sorrowful thoughts	0.80	0.788
		Melancholia	0.78	0.701
		Difficulty making decisions	0.73	0.582
Loss of vitality	0.885			
		Full of energy	0.77	0.727
		Physically fit and vital	0.69	0.741
		Enjoyed sexuality	0.65	0.507
		Calm and serene	0.65	0.701
		Happy	0.84	0.775
		Feeling healthy	0.75	0.760
Pain and cramps	0.809			
		Pain in the (lower) back	0.61	0.520
		Joint pain or pain in the limbs	0.72	0.703
		Muscle pain	0.76	0.716
		Neck or shoulder pain	0.70	0.553
		Muscle cramps	0.50	0.493
Neurovegetative symptoms	0.737			
		Trembling muscles	0.75	0.556
		Hot flushes or the chills	0.56	0.466
		Weakness	0.46	0.593
		Dizziness or a feeling that you might faint	0.56	0.523
		Diarrhea	0.65	0.425
Gastrointestinal symptoms	0.760			
		Nausea or upset stomach	0.73	0.613
		Abdominal pain or cramps	0.87	0.613

There was no difference in Cronbach's alpha in HPQ‐40 versus HPQ‐28. Cronbach's alpha ranged between 0.70 < α < 0.90.

Patients in all three patient groups with positive depression screening according to the included PHQ‐2 screening questions (score ≥3) also presented scores on the DaA scale significantly higher than those with negative screening (1.40 ± 0.62 versus 0.54 ± 0.54, *p* < 0.001), indicating the validity of this scale.

### 3.4 Revised version HPQ 28

The final version of the questionnaire, the HPQ 28, contains the 23 items represented on the five scales. From the 17 remaining questions not related to any of the five scales, the two items “heart palpitations or racing heart” and “numbness or tingling sensation in certain parts of the body” differed significantly between groups (*p* = 0.019 and *p* < 0.001) and were therefore included in the revised version (23 + 2 items). The item “troubled memory” was also included because cognitive impairment (sometimes described as “brain fog”) is often reported by patients with hypoparathyroidism and has been detected in several studies (23 + 2 + 1 items).^22^, ^23^ The screening questions for depression were included owing to the clinical relevance of depression in hypoPT patients but not as part of the DaA scale (23 + 2 + 1 + 2 = 28 items).

Patients with an inconsistent pattern in response were identified during the data analysis of the HPQ 40, indicating problems in the change from negatively to positively phrased questions. To avoid this bias in the revised version, positively phrased questions were positioned at the end of the questionnaire and new instructions were developed.

Furthermore, the font size for all items was increased to improve the legibility. In pretests, we determined that the time required to complete this shorter version was thus reduced to approximately 3 minutes. The questionnaire was both developed and tested in German. Items were translated into English by one person and then translated back into German by another person to validate the translation. Both translators assessed and clarified differences between the original and back‐translated German versions in wording, which was adjusted accordingly in the English version where necessary. The English version of the HPQ 28 is attached.

## 4 Discussion

Here we present a newly developed disease‐characteristic questionnaire for patients with hypoparathyroidism. Using a patient‐based analytical‐empirical approach, we identified five major symptom domains (scales) (depression and anxiety [DaA], loss of vitality [loss of VIT], pain and cramps [PaC], gastrointestinal [GIS], and neurovegetative symptoms [NVS]). The PaC, GIS, and NVS domains were newly identified and not expected on the basis of earlier studies. We conducted an initial investigation into the psychometric properties of this new questionnaire. This led to the “HPQ 40—Hypoparathyroid Patient Questionnaire” being revised and shortened accordingly to 28 items, followed by its renaming to HPQ 28.

Hypoparathyroid patients have a large number of different clinical symptoms.^4^, ^5^, ^7^, ^8^, ^9^ Our retrospective results in the self‐help groups compared with norms in the general population revealed increased depression and anxiety and reduced quality of life, thus confirming the results published in other studies.^4^, ^5^, ^7^, ^8^, ^9^

Taking the GBB as an example, increased somatic symptoms were found for heart complaints, pain in the limbs, and exhaustion. Hypoparathyroid patients have reported cardiac problems^6^, ^24^, ^25^ as well as fatigue and mental lethargy^12^ in the past. However, taking the tests as a whole, our results from the GBB, SF‐36, and SCL‐90 reveal that these tests are most likely not specific enough to depict the complaints of hypoparathyroid patients adequately enough, only demonstrating impairment in four of the five scales at best when compared with population norms. Moreover, patients added comments on some of the questionnaires without having been asked specifically to do so. These results strongly underline the need for a more specific test aimed at hypoparathyroid patients.

The different psychologic, somatic, and quality‐of‐life aspects of the symptoms were integrated into the new HPQ. To cover all relevant patients’ complaints, we systematically collected and (re)assessed patients’ comments with the “self‐help‐group‐derived questionnaire.” Usually, the members of self‐help groups experience a greater severity of illness^26^, ^27^ and patients suffering from disease more severely report more symptoms than patients with mild disease.^12^ Our new HPQ was therefore deemed likely to cover most of the relevant symptoms and complaints.

From a practical point of view, we prevented certain tendencies critical when designing the questionnaire. For example, we chose a four‐step scale to avoid the “error of central tendency” that occurs with an even number of responses without a neutral option in the center.^28^ Certain visual design elements were selected to structure the questionnaire on one single page to increase its acceptance by patients, nurses, and physicians alike. In addition, items were phrased in a manner of a symptom list, to make understanding both quick and easy. Not only is an appropriate questionnaire design of importance for patient compliance and understanding,^29^ but also a short questionnaire that is easy to understand and quick to complete is perhaps more likely to be implemented more regularly in clinical routine.^30^

A newly developed questionnaire is only accepted if it fulfills basic requirements such as practicability, feasibility, and low administrative burden, beyond the psychometric test criteria of reliability, validity, and objectivity.

With respect to the criteria for feasibility and administrative burden, the time required to complete the revised version is short for both the patient and the clinician: It takes approximately 3 minutes to fill in the HPQ 28. Results can be calculated automatically with provided SPSS syntax or manually in the office using a quick scoring list or transparent mask. Permission for clinical trials can be obtained and the material costs are low. The HPQ is thus feasible in daily work with a relatively low administrative burden.^31^, ^32^ Therefore, the acceptance of the questionnaire in clinical routine appears promising.

The objectivity of the HPQ is assured by standardized test instructions, predefined scaling, and analysis using standardized scoring instructions or SPSS syntax.

Reliability was measured with Cronbach's alpha as an indicator of internal consistency for each scale. The recommendation is that Cronbach's alpha lies between 0.7 and 0.9,^28^ which is the case for all of our five scales (PaC, GIS, loss of VIT, DaA, and NVS). The corrected item‐scale correlation represents the correlation of the individual item with the scale and needs to be greater than 0.3, which also applies to all items of the five scales, further supporting the reliability of the HPQ 28 questionnaire.

We started using a patient‐based approach to develop our questionnaire and subsequently added the input from clinicians. Prior and colleagues found that relevant symptoms reported by patients differed from those reported by clinicians, thus concluding that the experts’ view on disease does not necessarily match with the patients’ perception.^33^, ^34^ As Terwee and colleagues recommend, the target population needs to be involved in the item selection process to ensure content validity.^35^ Our analytical‐empirical methods as well as expert review further contribute to validity.

The identification of the five different scales for patients with hypoparathyroidism is of major importance. Studies on the symptoms of hypoparathyroid patients revealed various areas of complaints.^3^, ^4^, ^8^, ^12^ The HPQ questionnaire was therefore assumed to represent several dimensions of the disease. However, the number of factors (scales) was uncertain. As a result, exploratory factor analysis was performed that revealed five scales. Most items from the loss of VIT scale were originally influenced by the SF‐36 and therefore considered as representing an important aspect of patients’ quality of life.

Based on retrospective analysis and current knowledge from the literature, the two scales DaA and loss of VIT were expected to be relevant. The DaA scale was further validated by demonstrating its association with the PHQ‐2 items, which are known to have good sensitivity (79%) and specificity (86%) for detecting any depressive disorder,^36^ making it a suitable tool for screening. These PHQ‐2 items were integrated in our HPQ but were not included in the DaA scale. Patients from all groups screening positive through the PHQ‐2 questions also scored significantly higher on the DaA scale. This implies that our DaA scale measured their complaints appropriately. However, our DaA scale covers a broader symptom spectrum than the PHQ‐2 items alone.

The PaC, GIS, and NVS‐symptom‐based scales were newly identified through factor analysis. Even though muscle cramps and muscle pain are typical symptoms of hypoPT patients, these complaints are typically associated with decreased calcium levels. In addition, it was surprising that pain in the lower back, joint pain, pain in the limbs, as well as neck or shoulder pain not typically associated with hypoPT are main items on the scale for pain and cramps (PaC). As a result, further testing of HPQ 28, with an endorsed pain questionnaire, for example, is reasonable as a next step to validate these scales.^32^

Indeed, the identification of these new scales emphasizes the need for a disease‐characteristic questionnaire to reflect the problems of hypoparathyroid patients adequately.

The HPQ 28 has recently been translated into English to allow further testing with international patients. The English version was evaluated by back translation^28^ to identify potential differences in content and meaning. The few items not adequately back translated were discussed with both translators to find the best phraseology and wording judged to be the most suitable translation. This questionnaire can now be validated and employed in clinical trials, for example, with control groups and using longitudinal designs.

Limitations

As mentioned before, patients appeared to have problems with the change from positive to negative phrasing on the loss of VIT scale. Thus several structural changes had to be applied to improve patients’ understanding of this scale, and the instructions were clarified.^29^ Now, further testing in other patient cohorts is needed to confirm the improved comprehension of these important quality‐of‐life items.

The patient populations used in the development of the questionnaire (members of patient self‐help group and patients from two endocrinologic centers) were mostly women and their disease severity may differ from patients in general practice. Consequently, applying the questionnaire in different clinical settings and/or with patients of differing sex or age distributions will be of additional value.

Besides these minor limitations, the practical relevance and importance of a disease‐characteristic questionnaire in hypoparathyroidism is great, especially to identify, measure, and hence better understand the impairment caused by this disease. Further validation of the newly discovered symptom domains and standardization in larger populations are essential before the questionnaire can be implemented as a substitute for established generic questionnaires to quantify symptoms in patients with hypoparathyroidism. However, even now the HPQ 28 may be used to quantify and monitor symptom load in individual patients using the preliminary reference values. Studies involving larger groups of patients will reveal whether these preliminary reference values obtained from a relatively small sample can be generalized to patients with hypoparathyroidism from other settings or geographic regions.

In summary, we present a newly developed disease‐characteristic questionnaire for patients with hypoparathyroidism. Initial prospective testing revealed five major symptom domains with three newly identified scales. The HPQ 28—Hypoparathyroid Patient Questionnaire is now appropriate in the evaluation of hypoparathyroid patients’ complaints in clinical trials. The evaluation of its usefulness toward the quantitative measurement of characteristic symptoms and quality of life is of central interest to clinicians caring for patients with hypoparathyroidism.

## Disclosures

HS: advisory boards: MSD, Lilly, Amgen, Servier, Shire; speaker engagements: MSD, Lilly, Amgen, GSK, Servier, Shire. CH‐Li: advisory board: Pfizer; speaker: Servier, Heel, Novartis; royalties: Hogrefe Huber Publishers. All other authors state that they have no conflicts of interest.

## Supporting information

HPQ 28 – Questionnaire on Hypoparathyroidism
