# Supplementary material for: The HPQ—Development and First Administration of a Questionnaire for Hypoparathyroid Patients
Source: JBMR Plus. 2019 Nov 7;4(1):e10245. doi: 10.1002/jbm4.10245 (PMC6957982; doi:10.1002/jbm4.10245)
Supplement: Supplementary file 1 — HPQ 28 – Questionnaire on Hypoparathyroidism [file JBM4-4-e10245-s001.pdf]

HPQ 28 – Questionnaire on Hypoparathyroidism

Name/Number.:____ Date: ___________________

This questionnaire contains a list of questions about symptoms and complaints that can occur in connection with parathyroid gland insufficiency (hypoparathyroidism).

Please think about each question carefully and choose the answer that best fits, ticking the respective box.

Please answer every question.

In the past 4 weeks, how much did you suffer from....?	not at all	somewhat	quite a bit	severely
1. ...numbness or tingling sensation in certain parts of the body?	☐	☐	☐	☐
2. ...memory problems	☐	☐	☐	☐
3. ...pain in the lower back?	☐	☐	☐	☐
4. ...trembling muscles?	☐	☐	☐	☐
5. ...heart palpitations or racing heart?	☐	☐	☐	☐
6. ...joint pain or pain in the limbs?	☐	☐	☐	☐
7. ...self-blame?	☐	☐	☐	☐
8. ...nausea or upset stomach?	☐	☐	☐	☐
9. ...inner tension and restlessness?	☐	☐	☐	☐
10. ...abdominal pain or stomach cramps?	☐	☐	☐	☐
11. ...hot flushes or the chills?	☐	☐	☐	☐
12. ...muscle pain?	☐	☐	☐	☐
13. ...anxious thoughts?	☐	☐	☐	☐
14. ...neck or shoulder pain?	☐	☐	☐	☐
15. ...melancholia?	☐	☐	☐	☐
16. ... a sense of weakness?	☐	☐	☐	☐
17. ...dizziness or a feeling that you might faint?	☐	☐	☐	☐
18. ...difficulty making decisions?	☐	☐	☐	☐
19. ...diarrhea?	☐	☐	☐	☐
20. ...muscle cramps?	☐	☐	☐	☐

In the past 2 weeks, how often have you been bothered by any of the following problems	not at all	Several days	more than half the days	Nearly every day
21. Little interest or pleasure in doing things?	☐	☐	☐	☐
22. Feeling down, depressed or hopeless?	☐	☐	☐	☐

In the next section, we ask you some questions about your quality of life. Please note: the answer “not at all” in these questions indicates that your sense of well-being is highly impaired. The answer “strongly” thus is positive, meaning that you experienced only little or no impairment of your sense of well-being.

In the past 4 weeks, how much have you...	not at all	somewhat	quite a bit	strongly
23. ...felt full of energy?	☐	☐	☐	☐
24. ...felt physically fit and vital?	☐	☐	☐	☐
25. ...taken pleasure in sexuality?	☐	☐	☐	☐
26. ...been calm and serene?	☐	☐	☐	☐
27. ...been happy?	☐	☐	☐	☐
28. ...felt healthy?	☐	☐	☐	☐
